# Feral Pigeons (*Columba livia*) Prefer Genetically Similar Mates despite Inbreeding Depression

**DOI:** 10.1371/journal.pone.0162451

**Published:** 2016-09-02

**Authors:** Gwenaël Jacob, Anne-Caroline Prévot, Emmanuelle Baudry

**Affiliations:** 1 Ecologie Systématique Evolution, Univ. Paris-Sud, CNRS, AgroParisTech, Université Paris-Saclay, 91400, Orsay, France; 2 Department of Biology, University of Fribourg, Fribourg, Switzerland; 3 UMR7204, CNRS-MNHN-UPMC, Centre des Sciences de la Conservation (CESCO), Muséum National d’Histoire Naturelle, CP 51, 55 rue Buffon, Paris 5, France; Charles University in Prague, CZECH REPUBLIC

## Abstract

Avoidance of mating between related individuals is usually considered adaptive because it decreases the probability of inbreeding depression in offspring. However, mating between related partners can be adaptive if outbreeding depression is stronger than inbreeding depression or if females gain inclusive fitness benefits by mating with close kin. In the present study, we used microsatellite data to infer the parentage of juveniles born in a French colony of feral pigeons, which allowed us to deduce parent pairs. Despite detectable inbreeding depression, we found that pairwise relatedness between mates was significantly higher than between nonmates, with a mean coefficient of relatedness between mates of 0.065, approximately half the theoretical value for first cousins. This higher relatedness between mates cannot be explained by spatial genetic structure in this colonial bird; it therefore probably results from an active choice. As inbreeding but not outbreeding depression is observed in the study population, this finding accords with the idea that mating with genetically similar mates can confer a benefit in terms of inclusive fitness. Our results and published evidence suggest that preference for related individuals as mates might be relatively frequent in birds.

## Introduction

The pattern of occurrence and the consequences of inbreeding are of major interest in evolutionary and behavioural ecology, because inbreeding depression is considered to play a central role in the evolution of mating systems and dispersal [1–3]. Inbreeding, defined as mating among relatives, increases genome-wide homozygosity, which may be costly because it can decrease heterosis and unmask deleterious alleles in offspring [4], thereby reducing fitness. Such inbreeding depression has been frequently detected in natural populations of plants and animals [5]. Consequently, it is unsurprising that inbreeding avoidance is often reported and considered adaptive [6–8].

However, even in species in which inbreeding depression occurs it does not follow that mating among relatives should always be disfavoured. Firstly, when unrelated mating partners are scarce relative to related partners, the cost of inbreeding depression may be lower than that of forfeiting mating [9]. Secondly, too much outbreeding can also have negative fitness consequences for the offspring, because individuals in a population might carry coadapted gene complexes, making it maladaptive in terms of breaking them down, or because individuals might be adapted to local conditions [10,11]. Outbreeding depression therefore predicts “optimal outbreeding”: only first-order relatives should be avoided as mates to prevent close inbreeding; the optimal mate is a distant relative, not one from a distant population [12]. Finally, mating with a related partner could be advantageous, despite the adverse effect of inbreeding on offspring, because it increases the parent’s inclusive fitness [9,13,14]: an individual who mates with a relative will help that relative to spread genes identical by descent. Recent theoretical papers have shown that, under certain conditions, related mate preference is expected to evolve even when inbreeding depression is substantial if inclusive fitness is taken into account [15,16]; see [14] for a review.

Only a handful of empirical studies have reported mating preferences for related partners in animals (but see for example [17–19]), whereas inbreeding avoidance is considered to be the norm [6]. However Kokko and Ots [15] consider plausible that “the perception of ubiquitous inbreeding avoidance in nature follows from a mistaken view that it is the theoretical expectation”. Also, preference towards related mate might be difficult to demonstrate, because genetic variation is spatially structured in many species due to limited dispersal. If mated pairs are more related than random pairs, it is difficult to determine whether it is a consequence of a preference for related mates or of limited dispersal [20] in conjunction with a random mate choice with respect to relatedness. While it is possible to control for spatial genetic structure (e.g. [21]), it is usually difficult to determine the candidates available to a female when she is choosing a mate in a natural population. Therefore, to test this hypothesis, genetic similarity comparisons between mates and non-mates are now needed in species displaying a low genetic spatial structure

The rock pigeon, *Columba livia*, is well suited to perform such comparison. Firstly, this is a socially monogamous species [22]; secondly, genetic spatial structure exists in this species but, probably due to the high dispersal capacity of pigeons, differentiation is detectable only on a relatively large spatial scale (superior to about 20 km) and is entirely absent within a colony [23,24]. Thus, if the level of relatedness within breeding pairs deviates from the expectation under random mating, it must be the consequence of a mate choice process based on relatedness.

In the present study, we used microsatellite data to infer parentage of offspring produced in a dovecote located near Paris, France, allowing us to infer breeding pairs. We first tested the impact of within-pair relatedness on the number of offspring produced, to evaluate the fitness consequences of inbreeding in this population. We then compare the genetic relatedness of the inferred breeding pairs to the genetic relatedness expected under random mating, to determine whether relatedness influences mate choice in rock pigeon.

## Material and Methods

The study was carried out following the recommendations of the European Convention for the Protection of Vertebrate Animals used for Experimental and Other Scientific Purposes. Because the procedures used in the study (fitting with a colored ring and 1–3 μl blood draw) were minimally painful and because the pigeon *Columba livia* is considered a domestic species in France, ethical approval was not required by the French equivalent of the Institutional Animal Care and Use Committee (French "Direction Départementale des Services Vétérinaires"). The study was performed in only one location: a city owned dovecote. The study was authorized by the local authorities of the city (French "mairie" of Fontenay-sous-bois). The approval from the local authorities in Fontenay-sous-bois was obtained prior to sample collection.

### Study population

We studied a population of free-living pigeons (*Columba livia*) living in an urban dovecote in Fontenay-sous-Bois, France (48° 51’ N, 2° 28’ E). The dovecote is a small building (about 2.5 x 2.5 x 2.5 m) located in a park, where 90 nesting sites are available. Fifty kilograms of seeds are provided each week with grit and water. The pigeons have free access to the dovecote. Adults were captured either before sunrise, to catch the individuals that had spent the night in the dovecote, or in the daytime, to catch those using the dovecote mainly for feeding. A total of nine captures was performed over a 20-month period. Adults were forced out of the dovecote through a tissue tunnel and into cages placed on the floor. Juveniles were regularly captured in their nest, shortly before fledging, during the weekly maintenance of the dovecote. A total of 372 individuals (106 adult females, 120 adult males, and 146 juveniles) was captured. During each capture birds caught for the first time were fitted with a coloured ring. Our recapture data showed that the proportion of marked animals varied between 90 and 94% of the total number of pigeons. To perform parentage analyses (see below), we therefore conservatively assumed that 90% of parents were sampled.

### Genetic methods

From each individual, we sampled 1–3 μl of blood from the tarsal vein using a fine needle. Blood was stored in buffer PBS + EDTA (3% v/v) and kept frozen at -20°C until DNA extraction. We extracted DNA using the Qiagen (Hilden Germany) Tissue Kit and following the manufacturer’s protocol for DNA extraction from blood samples. DNA was eluted in 2x 100 μl AE buffer (Qiagen) and stored at -20°C. We amplified seven microsatellite loci developed for domestic breeds of the rock pigeon [25]. As feral pigeons show limited sexual dimorphism, a fragment of the chromo-helicase-DNA binding (CHD) gene was amplified to determine the sex of the individuals, following the method of Griffiths et al. [26]. F-primers were fluorescently labelled using the commercially available dyes, FAM, VIC, NED, and PET. Polymerase chain reactions (PCRs) were setup in 10μl volume containing 1x Multiplex Master Mix (Qiagen) and 0.2 μM each primer. We amplified the seven microsatellite loci in a single PCR and the CHD gene fragment in a second reaction. PCR conditions were as follows: 15 min at 94°C for DNA denaturation, 35 cycles of amplification [30 sec denaturation at 94°C, 3 min hybridisation at 54°C (48°C for molecular sexing), and 30 sec elongation at 72°C], and a final elongation step of 15 min at 72°C. The PCR products were mixed with an internal size standard (Genescan 500LIZ, Applied Biosystems) and analysed using an ABI 3700 semi-automated DNA sequencer (Applied Biosystems). We calculated the size of the microsatellite and CHD alleles using genescan 3.1 and genotyper 2.5 (Applied Biosystems). Negative controls were used to check for cross-contamination between tubes during DNA extraction and amplification.

One locus, CliμD19, showed a very high proportion of non-amplifying alleles (0.60) and was thus excluded from further analyses. The six other loci showed no evidence of heterozygote deficit, suggesting that null alleles, if present, were at low frequency (Table 1). Among the 372 DNA extracts analysed, 351 (94.3%) amplified at these six loci. The number of allele per locus ranged from 8 to 22 (mean number of alleles per locus = 14.5). The polymorphism information content (PIC) calculated with the program cervus 3.0 [27,28] was high, ranging from 0.602 to 0.869 (mean PIC = 0.784). We genotyped 96 individuals twice to estimate genotyping error rate (allelic dropout and false alleles). We observed only one difference at one locus for one individual between the replicates, i.e., a genotypic error rate of 1/(96x6) = 0.17%, a value within the normal range for genotypes obtained from invasive samples like blood [29]. The genotypes of the 351 individuals that were used in the parentage analyses (see below) are shown in S1 Table.

**Table 1:** Microsatellite statistics.

Locus	No. of alleles recorded	No. of individuals successfully genotyped	PIC	*H*_O_	*H*_E_	*F*(null)
CliμD17	8	377	0.602	0.668	0.647	0.000
CliμT17	10	377	0.781	0.798	0.809	0.006
CliμD16	22	373	0.788	0.804	0.809	0.000
CliμD32	15	374	0.869	0.850	0.882	0.018
CliμD01	22	373	0.853	0.874	0.867	0.000
CliμT13	10	363	0.810	0.793	0.833	0.024
Overall	14.5	377	0.784		0.808	

PIC, polymorphism information content; *H*o, level of observed heterozygosity; *H*e, level of expected heterozygosity; *F*(null), frequency of putative null alleles.

### Parentage analyses

Parentage analyses were conducted following the likelihood-based approach implemented in the program cervus 3.0 [27,28]. For each offspring, the program calculates the relative likelihood of parentage (LOD scores) for all possible parent pairs, thus allowing us to determine the most likely parent pair. Using allele frequency data from the population, the program then runs a simulation to estimate the critical LOD score value necessary for assignment at greater than 95% or 80% confidence levels. We used the following simulation parameters: 10,000 cycles, a proportion of missing values of 1% (value inferred from the data) and assuming that 90% of the parents were sampled (see above). We chose a genotypic error rate of 2% per locus to account for the occurrence of mistyping (estimated at a rate of 0.17%, see above), possible null alleles at low frequency, and mutations [28]. To evaluate the robustness of our results, we also performed analyses with slightly different values for the proportion of sampled parents (80 and 100%) and the error rate (1%).

Ten juveniles, four candidate mothers, and four candidate fathers were excluded from the analyses, because they did not amplify at one or more loci. Overall, 120 (88%) juveniles were assigned to a parent-offspring trio (38 and 82 at the 95% and 80% confidence levels, respectively). These assignments allowed us to infer 70 different parent pairs (13 pairs were inferred at least once from 95% confidence levels, while 57 pairs were inferred from 80% confidence levels). Some males and females contributed to more than one of the 70 different mother-father pairs inferred. In total, 51 (44%) of the 116 candidate males and 49 (48%) of the 102 candidate females reproduced, i.e., they were assigned as parent to at least one juvenile at 95 or 80% confidence levels. Very similar results were obtained when the proportion of sampled parents and the error rates were set to alternate values (not shown).

#### Reproductive success

Parentage was inferred for all offspring produced in the dovecote during the 20 months of the study. (Pairs that did not produce at least one juvenile during this period were therefore not included in this analysis.) Because not all adults were present in the dovecote during the 20 months and because we could not precisely evaluate the length of their stay in the dovecote, we measured the reproductive success of a parent pair as the number of juveniles that they produced by clutch. Feral pigeons lay one to two eggs per clutch. The reproductive success of the observed parent pairs was thus between one and two.

We used a generalized linear model with Binomial response to assess whether the probability of a pair having two offspring per clutch (given that the pair reproduced) was influenced by the relatedness between parents and by average monthly temperature and total precipitations during the time of incubation and the first weeks of growth of the chicks. All calculations were performed with the R statistical program using the “glm” function from the lme4 package [30].

Finally, we examined whether the breeding pairs observed in our study were on average more related than an equal number of pairs selected at random i) among all males and females present in the dovecote, and ii) among all males and females identified as breeders. We used the software program GenAlEx 6 [31] to calculate Queller and Goodnight’s *r* unbiased estimator of genetic relatedness [32]. This index is a measure of the degree to which the genetic similarity between two individuals exceeds the similarity between individuals randomly drawn from the population. Estimated relatedness values range from −1 to +1. Full siblings are expected to have a mean relatedness of 0.5 and unrelated individuals are expected to have a mean relatedness of 0.0 [33]. Negative values are observed when the allele frequencies of the two individuals differ from the population mean in opposite directions [32], i.e. they are less similar than expected by chance.

## Results and Discussion

### Genetic relatedness of couples and reproductive success

**Fig 1:**
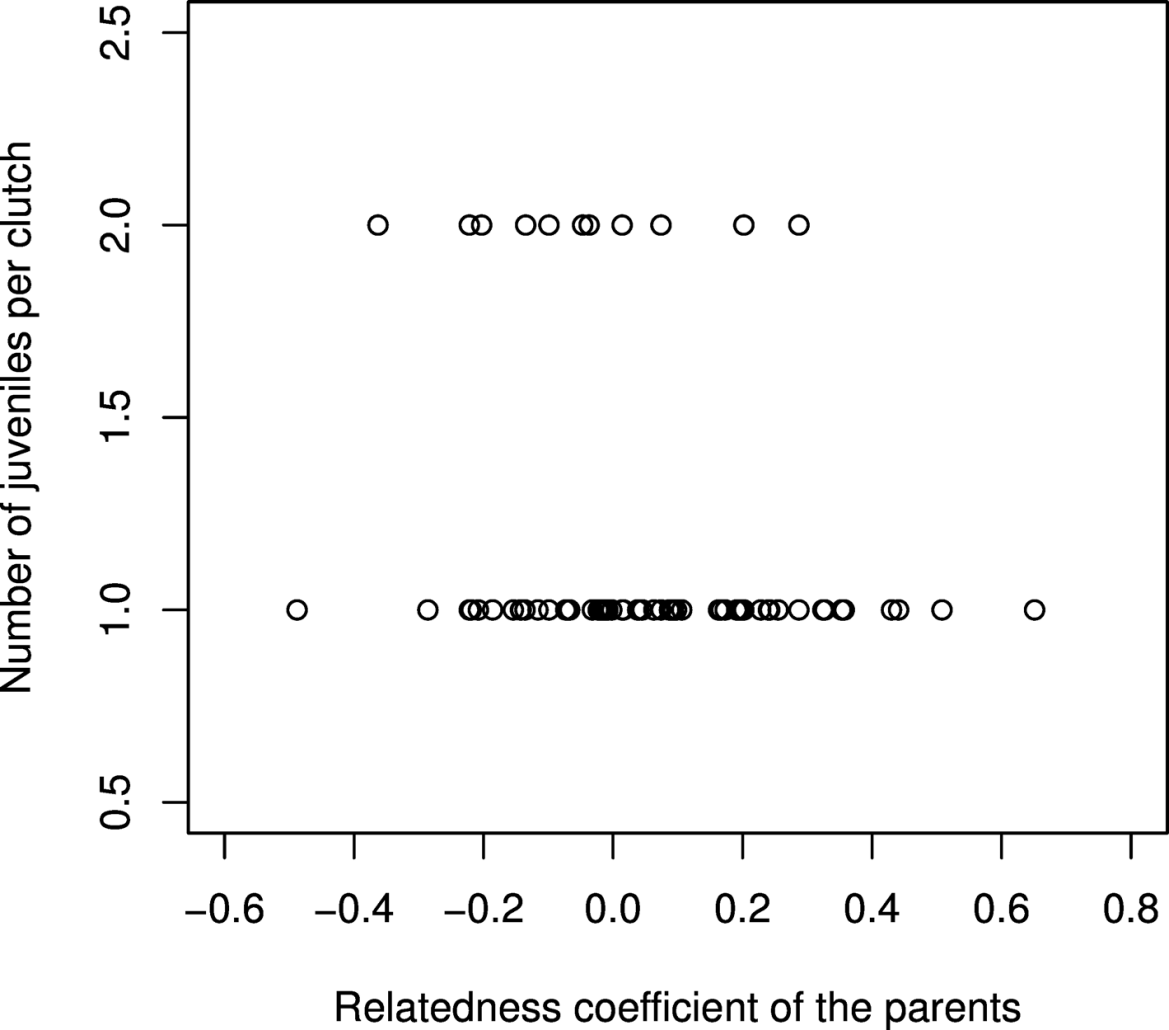
Genetic relatedness of couples and reproductive success, measured as the mean number of juvenile per clutch.

We studied whether the relatedness between mates had an influence on their reproductive success, measured as the number of juveniles per clutch (pairs that did not produce at least one juvenile were not included in this analysis). Mean temperature and total precipitations at the time of breeding were not retained during model selection based on AIC criteria, suggesting that breeding success is not affected by climatic conditions in our study population. We found that the probability of a pair having two offspring per clutch was negatively impacted by the level of relatedness within pairs (Estimate = -4.39, p-value = 0.006, null deviance = 91.91 on 101 degrees of freedom; Fig 1). This result indicates the existence of an inbreeding depression in our study population. A similar result was previously obtained in an experimental population of pigeons [34]; by performing controlled crosses, the authors of the study showed that parental relatedness does not affect egg production or hatching rate, but markedly decreases young viability. This accords with the observation that inbreeding depression seems to be a common phenomenon in natural bird populations (e.g. [5,8,35]).

### Genetic relatedness of couples

A total number of 70 effectively breeding couples were inferred from the parentage analyses, with several males and females belonging to more than one couple. The average pairwise relatedness of couples varied between −0.49 and +0.65, with a mean value of 0.062±0.22, being about half the theoretical value for first cousins (0.125). The average relatedness value was very close for couples inferred with a 95% (mean r = 0.063) or 80% (mean r = 0.061) confidence level.

We found that the 70 breeding pairs were on average more related that an equal number of pairs taken at random among all males and females present in the dovecote. The difference in level of relatedness between breeding pairs and random pairs was estimated at 0.07 and was highly significant (10,000 simulations, one-sided *p*-value = 0.0005).

A higher mate relatedness than expected by chance might be observed in the absence of mate choice based on relatedness if the adults that reproduce inside the dovecote do not form a representative sample of all adults present in the dovecote. For example, there could in fact be two genetically differentiated populations in the dovecote: adults reproducing in the dovecote and those using the dovecote only for feeding. To test this hypothesis, we compared the average relatedness between the 70 actual mates (0.062±0.22) and between an equal number of pairs selected at random among all males and females identified as breeders. We found that these random couples had a mean relatedness of -0.014±0.21. The difference in level of relatedness between observed pairs and random pairs was estimated at 0.10 and was highly significant (10,000 simulations, one-sided p-value < 0.0001). The abovementioned hypothesis can therefore be rejected.

Please note that we only tested the possibility that adults reproducing in the dovecote and those using the dovecote only for feeding were genetically differentiated. It remains theoretically possible that two genetically differentiated groups of pigeons reproduce in the dovecote but we think that it is unlikely. Feral pigeons have high dispersal abilities and at the spatial level, genetic structuration is detectable in urban pigeons only when populations are separated by more than 20 km [24]. Within an urbanized area, even a large one, feral pigeons show no genetic differentiation and behave as a single management unit [24,36]. Furthermore, we also calculated the inbreeding coefficient within the population. We found a low value (F_IS_ = 0.009), which confirms that no genetic differentiation is present within the population. Finally, to our knowledge, genetic structure has never been detected within a colony in any species of colonial bird. We therefore think that the presence of two genetically differentiated groups of pigeons within the dovecote is unlikely.

A higher relatedness between mates might also have been observed in our study, because couples were inferred from their offspring: if mate choice is not dependent on relatedness but rather if related couples have a better reproductive output on average, then this could result in the observed pattern. However, our data clearly show that the opposite is true: related couples tend to have lower reproductive success. Yet as we did not consider pairs that failed to reproduce, we probably underestimated the average relatedness between mates. We therefore conclude that feral pigeons probably choose genetically similar individuals as mates, thus increasing inbreeding levels in the population. This is in agreement with the findings of a study on the spatial genetic structure of feral pigeon populations in France: the large majority of the studied populations showed positive inbreeding coefficient (F_IS_) values [24], suggesting the presence of inbreeding.

The first question that deserves discussion is the mechanism that leads to the observed level of inbreeding. Genetic similarity between mates can be observed even if mate choice is random with respect to kinship in the presence of a small-scale genetic population structure. In such a case, encountered potential mates are more genetically similar than random mates from the population (see, for example, the case of a blue tit population [37]). However, here we observed that the pigeon mates inhabiting a single dovecote were more genetically similar than random pairs, excluding spatial genetic structure as a possible cause. This suggests that the genetic similarity between mates could be caused by kin-based mate choice. There is a large body of evidence for kin recognition in birds, mostly based on the associative learning of visual and acoustic cues (reviewed in [38]). Recently, it was shown that olfactory clues alone can also allow birds to discriminate between kin and non-kin [39]. Interestingly, while kin recognition is usually thought to allow the avoidance of related mates, here it could be used to favour them.

The second question concerns the fitness consequences of the level of inbreeding that we observed in the population (0.062), i.e., what might be the selective pressure responsible for the evolution of a kin preference? Given that the widespread low level of inbreeding observed in this population seems to be the result of an active choice, it seems likely that this level of inbreeding would be beneficial. A first possibility would be that the preference for mates of intermediate genetic similarity observed in our study population leads to optimal outbreeding [40]. However, our data clearly indicates that inbreeding negatively impacts reproductive success in our study population. A recent meta- analysis [41] found a positive relationship between the relatedness of social mates and the occurrence of extrapair paternity (EPP) in birds. EPP might thus be a strategy to help in mitigating the potential negative effects of pairing with a genetically similar mate, suggesting again that inbreeding is usually harmful. Furthermore, within a natural population outbreeding depression is usually thought to be negligible except when individuals mate with immigrants from distant populations [8], which is not the case here. The optimal outbreeding hypothesis seems therefore not to apply to the study population.

A second hypothesis would be that mating with a related partner can be advantageous, not because it maximises the offspring’s fitness, but because it increases the parent’s inclusive fitness [13]. For this reason, inbred mating may be favoured even in the presence of a substantial inbreeding depression. Kokko and Ots [15] showed that inbred mate preference may evolve even when the inbreeding depression substantially exceeds 1/3. Within our study population, the preference for genetically related mates decreases the probability of having two offspring by 21.8%, (note however, that because couples were inferred from their offspring, couples that did not produce fledging juveniles were not detected, which probably results in an underestimation of the inbreeding depression). More recently, Puurtinen [16] showed that mating with intermediately related individuals maximizes inclusive fitness for wide range of realistic inbreeding depression strengths (but see [42]). It therefore seems likely that in pigeons, the increase in indirect fitness conferred by mating with a related partner more than outweighs the decrease in direct fitness due to inbreeding depression.

Experimental choice studies in a variety of species have demonstrated that close relatives are usually avoided as mating partners [6]. Yet inbreeding depression seems to be pervasive in natural populations [5], offering a simple adaptive explanation for this behaviour. Consequently, the avoidance of relatives as mates has almost become a paradigm of mating system theories. However, the same experimental studies often reported that if first-order relatives are usually avoided as mates, intermediate relatives (for example, first or second cousins) are frequently preferred over unrelated individuals [6]. Furthermore, the development of molecular techniques has recently allowed us to more easily study the relatedness of mates in natural populations, with contrasting results. We reviewed the studies that used molecular or pedigree data to compare the average relatedness observed between mates with the relatedness expected under random mating in wild populations of single breeding birds (Table 2, adapted and updated from [8]). In most cases, a random process of mate choice with respect to relatedness was observed (Table 2). However, preference for related mates was detected relatively often, in five out of 17 studies, even if it was restricted to specific conditions in two out of the five cases. Finally, mates were on average less related than expected under random mating in only two cases. In conclusion, it seems that the preference for genetically similar mates that we observed in pigeons is not exceptional. Our results and published evidence suggest that preference for relatives as mates might be more frequent than initially thought.

**Table 2:** A list of studies using molecular or pedigree data to compare the average relatedness observed between mates with the relatedness expected under random mating in wild populations of birds. Adapted and updated from Table IV of Kempenaers (2007).

Species^a^	Mate choice	Comments	Study
Barn swallow, *Hirundo rustica*	Random	Social pairs versus random dyads	[43]
	Prefer related mate	Extra pairs versus random dyads	[43]
Black-legged kittiwake, *Rissa tridactyla*	Avoid related mate	Pairs versus random dyads	[44]
Blue tit, *Parus caeruleus*	Prefer related mate	Social pairs versus random dyads (island)	[45]
	Random	Social pairs versus random dyads (mainland)	[45]
Blue tit, *Parus caeruleus*	Random	Social pairs and extra pairs vs. random dyads	[36]
Feral pigeon, *Columba livia*	Prefer related mate	Pairs versus random dyads	This study
Great tit, *Parus major*	Random	Pairs versus nearest neighbour	[21]
Great tit, *Parus major*	Random	Pairs versus nearest neighbour	[46]
	Random	Pairs versus random male in the same patch	[46]
Great frigate, *Fregata minor*	Prefer related mate	Pairs versus random male (island)	[17]
Great reed warbler, *Acrocephalus arundinaceus*	Random	Pair versus random candidate mates	[47]
House finch, *Haemorhous mexicanus*	Random	Early pairs	[46]
	Avoid related mate	Late pairs	[46]
House sparrow, *Passer domesticus*	Prefer related mate	Social and extra pairs versus random dyads (island)	[19]
House sparrow, *Passer domesticus*	Random	Pairs versus random dyads	[48]
House sparrow, *Passer domesticus*	Random	Also compared to nonbreeding males	[49]
NZ robin, *Petroica australis*	Random	Pairs versus random dyads	[50]
NZ saddleback, *Philesturnus carunculatus*	Random	Pairs versus random dyads	[50]
Satin bowerbird, *Ptilonorhynchus violaceus*	Random	Pairs versus random candidate mates	[51]
Savannah sparrow, *Passerculus sandwichensis*	Random	Analysed separately over 4 years	[52]
Song sparrow, *Melospiza melodia*	Random	Pair versus three sets of potential mates	[53]

^a^As it is usually more difficult to assess potential mates in cooperative breeding species, we restricted this list to single breeding species.

## Supporting Information

S1 Table Genotypes of the 351 individuals that were used in the parentage analyses. (XLSX)

## References

[1] Brooker M, Rowley I, Adams M, Baverstock P. Promiscuity: An inbreeding avoidance mechanism in a socially monogamous species? Behav Ecol. 1990; 26: 191–199.

[2] Greenwood PJ, Harvey PH, Perrins CM. Inbreeding and dispersal in the great tit. Nature. 1978; 271: 52–54. 10.1038/271052a0

[3] Wolff JO. Parents suppress reproduction and stimulate dispersal in opposite-sex juvenile white-footed mice. Nature. 1992; 359: 409–410. 10.1038/359409a0 1406952

[4] Charlesworth D, Willis JH. The genetics of inbreeding depression. Nat Rev Genet. 2009; 10: 783–796. 10.1038/nrg2664 19834483

[5] Keller L, Waller D. Inbreeding effects in wild populations. Trends Ecol Evol. 2002; 17: 230–41. 10.1016/S0169-5347(02)02489-8

[6] Pusey A, Wolf M. Inbreeding avoidance in animals. Trends Ecol Evol. 1996; 11: 201–206. 21237809 10.1016/0169-5347(96)10028-8

[7] Amos W, Wilmer JW, Fullard K, Burg TM, Croxall JP, Bloch D, et al The influence of parental relatedness on reproductive success. Proc Biol Sci. 2001; 268: 2021–2027. 10.1098/rspb.2001.1751 11571049 PMC1088844

[8] Kempenaers B. Mate choice and genetic auality: A review of the heterozygosity theory. Adv Study Behav. 2007; 37: 189–278. 10.1016/S0065-3454(07)37005-8

[9] Waser PM, Austad SN, Keane B. When should animals tolerate inbreeding? Am Nat. 1986; 128: 529–537. 10.1086/284585

[10] Bateson P. Sexual imprinting and optimal outbreeding. Nature. 1978; 273: 659–660. 10.1038/273659a0 661972

[11] Frankham R, Ballou JD, Eldridge MDB, Lacy RC, Ralls K, Dudash MR, et al Predicting the probability of outbreeding depression. Conserv Biol. 2011; 25: 465–475. 10.1111/j.1523-1739.2011.01662.x 21486369

[12] Bateson P. Preferences for cousins in Japanese quail. Nature. 1982; 295: 236–237. 10.1038/295236a0

[13] Bengtsson B. Avoiding inbreeding: At what cost? J Theor Biol. 1978; 73: 439–444. Available from: http://www.sciencedirect.com/science/article/pii/0022519378901510 692150 10.1016/0022-5193(78)90151-0

[14] Szulkin M, Stopher K V., Pemberton JM, Reid JM. Inbreeding avoidance, tolerance, or preference in animals? Trends Ecol Evol. 2013; 28: 205–211. 10.1016/j.tree.2012.10.016 23182684

[15] Kokko H, Ots I. When not to avoid inbreeding. Evolution. 2006; 60: 467–475. 10.1111/j.0014-3820.2006.tb01128.x 16637492

[16] Puurtinen M. Mate choice for optimal (k)inbreeding. Evolution. 2011; 65: 1501–1505. 10.1111/j.1558-5646.2010.01217.x 21521199

[17] Cohen LB, Dearborn DC. Great frigatebirds, *Fregata minor*, choose mates that are genetically similar. Anim Behav. 2004; 68: 1229–1236. 10.1016/j.anbehav.2003.12.021

[18] Schjørring S, Jäger I. Incestuous mate preference by a simultaneous hermaphrodite with strong inbreeding depression. Evolution. 2007; 61: 423–430. 10.1111/j.1558-5646.2007.00028.x 17348951

[19] Bichet C, Penn DJ, Moodley Y, Dunoyer L, Cellier-Holzem E, Belvalette M, et al Females tend to prefer genetically similar mates in an island population of house sparrows. BMC Evol Biol. 2014; 14:47–58. 10.1186/1471-2148-14-47 24621140 PMC3984696

[20] Bohonak A. Dispersal, gene flow, and population structure. Q Rev Biol. 1999; 74: 21–45. 10081813 10.1086/392950

[21] Szulkin M, Zelazowski P, Nicholson G, Sheldon BC. Inbreeding avoidance under different null models of random mating in the great tit. J Anim Ecol. 2009; 78: 778–788. 10.1111/j.1365-2656.2009.01544.x 19383076

[22] Johnston RF, Janiga M. Feral pigeons Oxford: Oxford University Press; 1995.

[23] Kimura M, Sano A, Sobue H. Genetic variability within and between feral pigeon populations. Research Bulletin of the Faculty of Agriculture, Gifu University. 1991; 56: 7–13.

[24] Jacob G, Prévot-Julliard AC, Baudry E. The geographic scale of genetic differentiation in the feral pigeon (*Columba livia*): Implications for management. Biol Invasions. 2015; 17: 23–29. 10.1007/s10530-014-0713-2

[25] Traxler B, Brem G, Muller M, Achmann R. Polymorphic DNA microsatellites in the domestic pigeon, *Columba livia* var. domestica. Mol Ecol. 2000; 9: 366–368. 10.1046/j.1365-294x.2000.00874-2.x 10736035

[26] Griffiths R, Double M, Orr K, Dawson RJ. A DNA test to sex most birds. Mol Ecol. 1998; 7: 1071–1075. Available from: http://onlinelibrary.wiley.com/doi/10.1046/j.1365-294x.1998.00389.x/pdf 9711866 10.1046/j.1365-294x.1998.00389.x

[27] Kalinowski ST, Taper ML, Marshall TC. Revising how the computer program CERVUS accommodates genotyping error increases success in paternity assignment. Mol Ecol. 2007; 16: 1099–1106. 10.1111/j.1365-294X.2007.03089.x 17305863

[28] Marshall T. Cervus Version 2.0 Edinburgh: University of Edinburgh; 2001.

[29] Hoffman J, Amos W. Microsatellite genotyping errors: Detection approaches, common sources and consequences for paternal exclusion. Mol Ecol. 2005; 14: 599–612. 15660949 10.1111/j.1365-294X.2004.02419.x

[30] Pinheiro J, Bates D, DebRoy S, Sarkar D, Team RC. nlme: Linear and nonlinear mixed effects models. R package version 3.1–90. 2008.

[31] Peakall R, Smouse P. GenAlEx 6.5: Genetic analysis in Excel. Population genetic software for teaching and research—an update. Bioinformatics. 2012; 28: 2537–2539; Available from: http://bioinformatics.oxfordjournals.org/content/28/19/2537.short 22820204 10.1093/bioinformatics/bts460 PMC3463245

[32] Queller DC, Goodnight KF. Estimating relatedness using genetic markers. Evolution. 1989; 43: 258–275. 10.2307/2409206 28568555

[33] Blouin M, Parsons M, Lacaille V, Lotz S. Use of microsatellite loci to classify individuals by relatedness. Mol Ecol. 1996; 5: 393–401. 8688959 10.1111/j.1365-294x.1996.tb00329.x

[34] Soulaymani B, Mokhtari B. Impact du degré de parenté sur la prolificité, l’éclosabilité et la viabilité des descendants dans une population expérimentale de pigeons. J Anim Breed. 1999; 116: 139–150. Available from: http://onlinelibrary.wiley.com/doi/10.1046/j.1439-0388.1999.00182.x/full

[35] Bensch S, Hasselquist D, Schantz T von. Genetic similarity between parents predicts hatching failure: Nonincestuous inbreeding in the great reed warbler? Evolution. 1994; 48: 317–26. 28568307 10.1111/j.1558-5646.1994.tb01314.x

[36] Giunchi D, Albores-Barajas YV, Baldaccini NE, Vanni L, Soldatini C. Feral pigeons: problems, dynamics and control methods. In: Larramendy ML, Soloneski S, editors. Integrated pest management and pest control—current and future tactics. InTech; 2012. pp 215–240.

[37] Foerster K, Valcu M, Johnsen A, Kempenaers B. A spatial genetic structure and effects of relatedness on mate choice in a wild bird population. Mol Ecol. 2006; 15: 4555–4567. 10.1111/j.1365-294X.2006.03091.x 17107482

[38] Nakagawa S, Waas JR. O sibling, where art thou? A review of avian sibling recognition with respect to the mammalian literature. Biol Rev. 2004; 79: 101–119. 10.1017/S1464793103006249 15005175

[39] Krause ET, Kruger O, Kohlmeier P, Caspers BA. Olfactory kin recognition in a songbird. Biol Lett. 2012; 8: 327–329. 10.1098/rsbl.2011.1093 22219391 PMC3367747

[40] Bateson P. Optimal breeding In: Bateson P, editor. Mate choice. Cambridge, UK: Cambridge University Press; 1983 p. 257–277.

[41] Arct A, Drobniak SM, Cichoń M. Genetic similarity between mates predicts extrapair paternity—a meta-analysis of bird studies. Behav. Ecol. 2015; arv004. 10.1093/beheco/arv004

[42] Cherry J. L Inbreeding and asexuality: a response to Szulkin et al Trends Ecol Evol. 2013; 28: 683 10.1016/j.tree.2013.08.001 24189156

[43] Kleven O, Jacobsen F, Robertson RJ, Lifjeld JT. Extrapair mating between relatives in the barn swallow: A role for kin selection? Biol Lett. 2005; 1: 389–392. 10.1098/rsbl.2005.0376 17148214 PMC1626374

[44] Mulard H, Danchin E, Talbot SL, Ramey AM, Hatch SA, White JF, et al Evidence that pairing with genetically similar mates is maladaptive in a monogamous bird. BMC Evol Biol. 2009; 10.1186/1471-2148-9-147 PMC2709659 19566922

[45] Lifjeld JJ, Krokene C. Variation in the frequency of extra-pair paternity in birds: A comparison of an island and a mainland population of blue tits. Behaviour. 2000; 137: 1317–1330. 10.1163/156853900501944

[46] van de Casteele T, Matthysen E. Natal dispersal and parental escorting predict relatedness between mates in a passerine bird. Mol Ecol. 2006; 15: 2557–2565. 10.1111/j.1365-294X.2006.02946.x 16842426

[47] Hansson B, Jack L, Christians JJK, Pemberton JM, Åkesson M, Westerdahl H, et al No evidence for inbreeding avoidance in a great reed warbler population. Behav Ecol. 2007; 18: 157–1564. 10.1093/beheco/arl062

[48] Edly-Wright C, Schwagmeyer PL, Parker PG, Mock DW. Genetic similarity of mates, offspring health and extrapair fertilization in house sparrows. Anim Behav. 2007; 73: 367–78. 10.1016/j.anbehav.2006.08.008

[49] Bonneaud C, Chastel O, Federici P, Westerdahl H, Sorci G. Complex Mhc-based mate choice in a wild passerine. Proc Biol Sci. 2006; 273: 1111–6. 10.1098/rspb.2005.3325 16600889 PMC1560269

[50] Jamieson IGI, Taylor SSS, Tracy LLN, Kokko H, Armstrong DP. Why some species of birds do not avoid inbreeding: Insights from New Zealand robins and saddlebacks. Behav Ecol. 2009; 20: 575–84. 10.1093/beheco/arp034

[51] Reynolds SM, Uy JAC, Patricelli GL, Coleman SW, Braun MJ, Borgia G. Tests of the kin selection model of mate choice and inbreeding avoidance in satin bowerbirds. Behav Ecol. 2014;, aru065.

[52] Freeman-Gallant CR, Wheelwright NT, Meiklejohn KE, Sollecito SV. Genetic similarity, extrapair paternity, and offspring quality in Savannah sparrows (*Passerculus sandwichensis*). Behav Ecol. 2006; 17: 952–8. 10.1093/beheco/arl031

[53] Keller LF, Arcese P. No evidence for inbreeding avoidance in a natural population of song sparrows (*Melospiza melodia*). Am Nat. 1998; 152: 380–92. 10.1086/286176 18811446

